# Preventive health behaviors and physician visits: relevance to health inequality

**DOI:** 10.1186/s13584-015-0006-y

**Published:** 2015-03-13

**Authors:** Varda Soskolne

**Affiliations:** The Louis and Gabi Weisfeld School of Social Work, Bar Ilan University, Ramat Gan, 52900 Israel

**Keywords:** Preventive health behaviors, Physician visits, Health inequalities

## Abstract

Clear definitions and measurement of preventive health behaviors, as well as the relevant demographic and socioeconomic variables, is important to understanding what factors explain inequalities in health and in the use of health care services. This commentary addresses issues related to the measurement of preventive health behaviors and suggests a distinction between personal life style behaviors and preventive screening practices in order to better explain the associations between these practices and visits to general practitioners.

The commentary notes that physician visits are a health-related behavior which is shaped by socioeconomic status: visits to general practitioners are more prevalent among the poor, while visits to specialists are more prevalent among the rich. Therefore, in any analysis of the factors contributing to socioeconomic inequalities in health, physician visits and preventive health behaviors ought to be included as two distinct sets of health-related behaviors. Changing these health-related behaviors is only one of the interventions that are better developed by healthcare services, while the majority of multi-level efforts to reduce inequalities should be outside of the health sector.

Access to comprehensive, quality health care services is important for the achievement of health equity and for increasing the quality of a healthy life for everyone. Yet, even when access to services may be equitable, under a universal health care system, utilization of health services is usually unequal, being one of the factors affected by the social determinants of health – the economic and social conditions of daily living that determine a person’s chances of maintaining good health [1]. These conditions are influenced by policy choices and are shaped by the amount of money, power, and psychosocial resources that people have, and affect the physical environment, and the person’s individual characteristics and behaviors [1]. People with greater resources are better able to use health care services in order to improve their health than people with fewer resources. Inequalities in the use of health care services enhance the risk of disease and increase social inequalities in health [2].

Within a large body of research on social inequalities in health, the study conducted by Yom Din, Zugman and Khashper [3] looked at utilization of medical services as the outcome of interest. The authors examined the joint effect of preventive health behaviors (PHBs) and socio-demographic factors on the utilization of medical services, measured by visits to either a General Practitioner (GP) or a Specialist Doctor (SD). This secondary data analysis of the 2009 Israeli National Health Survey of 8,713 households, covering 28,968 individuals demonstrates that PHBs have a positive and highly significant effect on visits to both types of doctors, together with significant associations of the socio-demographic variables (socio-economic status, marital status, age and living with a chronic disease). Of these, a major finding reflects inequalities in utilization of GP and SD by socio-economic status (SES) (pro-poor for visits to GP, pro-rich for visits to SD.

The findings add important information to several areas of research and have significant implications for practice and policy. They shed updated light on social inequalities in health services utilization in Israel, particularly inequalities by SES. The important and novel contribution of the article is the examination of the unique role of PHBs relative to that of social-demographic factors, in influencing visits to primary and specialist care services. It does so by addressing a methodological issue – that of endogeneity. Thus, the findings allow discussion of issues of measurement of PHBs and of conceptualization of both PHBs and physician visits, as well as the implications for their relevance as explanatory factors in the pathways between social determinants of health and health outcomes.

There is a consistent and large body of evidence of the substantial effect that SES has on the health of individuals [4,5] and on the utilization of health services [6]. While the Yom Din et al.’s findings on the SES inequalities in visits to the doctors are in line with previous evidence from studies in Israel [7], it is of interest to see how these data compare to those in other OECD countries, some of them with comparable universal health care system. Shmueli [8] showed that after adjusting for health needs, the Israeli poor, relatively to the rich, enjoy more family doctors’ services than in all other countries, while the pro-rich inequality for visits to a SD locates Israel at the center of the range, with pro-rich inequality lower than that in Canada, Switzerland and France. One explanation, as Shmueli argues, is the zero (or very low)-copayment for family doctors visits in Israel, and the relatively low copayment for specialist care in Israel. Nevertheless, other factors underlying physician visits beyond cost should not be ignored. People go to a clinic not only to obtain curative or preventive services, (i.e., the manifest functions of the services) but also to satisfy other, nonmedical latent functions, such as legitimation of failure, to gain an accepting listener for cathartic needs, or to obtain other “secondary benefits”, including sick leave [9]. People with lower SES may feel more comfortable communicating about their health problems with the GP than with a specialist, as the former may be more understanding of their personal needs and their family context, while patients with a higher SES may trust and seek a ‘more specialized’ provider [6]. This may be one of the reasons why GP services are associated with pro-poor utilization while SD services are associated with pro-rich utilization. . It should also be noted that while individuals may self-refer to a GP, specialty health services usually require referral from a GP who operates as a gatekeeper for secondary health services utilization. Information regarding the reasons for the visit to a GP (care or request for referral) or the referral source to a specialist (self or GP referral) which is lacking in most studies, limits a more refined understanding of the differences in SES inequalities in visiting GP or SD.

Similar to Yom Din et al.’s findings, other recent data in Israel showed that persons with low SES are less likely to engage in preventive practices despite their higher use of GP services [10]. Yom Din et al. argue that there are bidirectional influences of PHBs and visiting a doctor: various PHBs influence the use of medical care and PHBs can be influenced by visiting a doctor - by receiving recommendation to adopt health practices - and thus the problem of endogeneity between medical care utilization and PHBs needs to be addressed. Although this is an important method, validity of the measures should not be automatically assumed.

A PHB index was defined in the study as a combination of “good health practices”. It was constructed as equally weighted scores of one life style behavior (physical activity, after exclusion of smoking) and one or two screening behaviors (flu vaccination for both men and women and the addition of mammography screening for women aged 50–74). Under the assumption that there does not seem to be reciprocal influence between visits to a SD and these behaviors, endogeneity was tested for GP visits only and was confirmed. Yet, is this sufficient to minimize a threat to validity of the PHB measure in this study? The combination of behaviors implies that physical activity, a personal health promoting behavior is judged as equal to preventive practices (flu vaccination, mammography screening). The former behavior is dependent on the individual’s motivation and continuous determination, even if recommended by a GP, and is time consuming. The latter behaviors are usually performed following a recommendation of the GP or a reminder from the health care provider, are limited in time, and (at least for mammography) require a referral from a GP. It could be that the screening practices are the source of endogeneity to a greater extent than that of life style behaviors. Separate examinations of the two types of preventive health behaviors may provide evidence to better guide targeted interventions. For example, efforts at improving quality and addressing inequalities made by HMO’s in Israel, have led to substantial reductions of the SES gaps in screening procedures (such as mammography [10]), while inequalities in life style behavior decreased only slightly (e.g. the ratio of low vs. high education in physical activity was 0.5 in 2014 [10] and somewhat under 0.6 in 2010 [7]). Inclusion of smoking among the personal health behaviors may produce different results regarding endogeneity, as one recent study demonstrated that smoking *reduces* the probability of using health care services [11], an opposite direction to that found with the Yom Din et al.’s index which exclude smoking. The differentiation of PHBs may be particularly relevant for future studies in view of the emerging changes in modes of and reasons for use of GPs, as various forms of virtual visits - telehealth and e-visits are increasing, which subsequently may decrease office visits.

Another aspect relates to the conceptualization of PHBs and GP visits. Visiting a doctor is also a behavior. The most widely-used model of use of services – the Andersen’s Behavioral Model of use of health care services - conceptualizes behavior as a function of people's predisposing characteristics (i.e. age, gender, beliefs), their enabling characteristics – the personal and community resources (i.e. SES, marital status, accessibility of services, etc.), and individual needs (i.e. objective and subjective health status) [12]. Moreover, in the revised Behavioral Model which puts health status as the final outcome, personal health practices such as diet, exercise, and self-care are included separately as interacting with use of formal health services as two sets of behaviors that influence health outcomes [12]. Thus, differentiating personal health behaviors from preventive screening behaviors as a further refinement, and together with the behavioral aspect of use of services may provide better estimates of their relative contribution to health as a means of reducing health inequalities.

Health behaviors and utilization of health services are only part of the wider array factors included in various approaches for explaining health inequalities. Yet, there is a consensus that social position is linked to health via complex multilevel pathways. The social structure macro-level factors impact on the social and cultural environment and on the organization of health and welfare systems which exert their influence on individual level material, psychosocial, or community factors, thereby linking SES to health-related behaviors and to biological response [13]. While health-related behaviors are the most proximate to health outcomes, some of the psychosocial factors, (lack of) social support for example, are known to be as detrimental to health as many forms of risky behavior [14]. Therefore, without acknowledging the external environment as an important input for understanding use of health services [12], analyses of the SES associations with PHBs and with utilization of GP and SD provide only partial basis for practice. One indication comes from Terraneo’s comparative study in 12 European countries [11]. Characteristics of the healthcare system seem to be limited in moderating the educational inequalities in utilization of healthcare services, but in countries with higher total expenditure on health, more educated people tend to reduce specialist visits and increase GP care.

The theoretical framework of the social determinants of health was supported by empirical findings and recommended that a combination of macro and individual specific factors in each country become the basis for policy aimed at tackling health inequalities [1]. Many interventions have mainly focused on changing health behaviors. There is a consensus that health behaviors, risky or preventive practice, are particularly relevant to health inequalities: if behavior could be changed - whether by individual approaches or by wider social interventions - inequalities might be alleviated. Yet, an in-depth review concluded that interventions designed to change behavior rarely alleviate inequalities in health, and in some cases may exacerbate them [15]. More rigorous evidence is necessary, but it is also important to note that ignoring other factors may lead to partial success in reducing health inequalities.

This commentary does not intend to detail successful interventions, a topic for another article, but to conclude by affirming the role of healthcare systems in tackling health inequalities. Inequality stems to a large extent from socioeconomic differences that exist within a society. Israel is among the Organization for Economic Co-operation and Development (OECD) countries with the highest income inequality, measured by the Gini coefficient [16], reflecting societal values and policy decisions that also shape the structure, funding and provision of healthcare systems. While the majority of efforts to reduce inequalities should be outside of the health sector, healthcare services can still have a large impact on SES inequalities in health if they also reorient toward the basic determinants of health and develop and implement interventions adapted to the needs of disadvantaged groups in their own society. This understanding has already guided national or regional programs aimed at reducing health inequalities, including that of the Israel Ministry of Health which selected it as one of its strategic aims [17]. This was also the approach adopted by members of the subcommittee on health of the Elalouf Poverty-Fighting Committee. The Committee was appointed in November 2013 by the Israel Minister of Social Affairs, in an attempt to respond to the alarmingly high rate of poverty in Israel, nearly twice the OECD average of 11.3% [16]. The overall goal of the Committee was to reduce poverty rates to the OECD average within ten years by making overarching and specific recommendations in five major areas: welfare and allowances, housing, education, employment and health. A separate sub-committee was appointed to address each major area. The recommendations of the sub-committee on health (which I had the privilege to chair) focused on ways to decrease the risk to health due to poverty and to promote the health status of people living in poverty by several measures, some of which particularly targeted the pro-rich disparity in visits to SDs by recommending exemptions or discounts of co-payments for SDs visits and medications [18]. Unfortunately, lack of political will is a major reason for failing to deal with the health inequalities in a more integrated program in cooperation with other government offices. None of the recommendation regarding health has been adopted to date and only a tiny part of other subcommittees’ recommendations have been budgeted by the government. Similar effects of political decisions on reducing health inequalities in other countries have brought Ilona Kickbusch [19] to call upon public health professionals to further explore the political determinants of health.
